# Prevalence of serum *Aspergillus* galactomannan positivity among immunocompromised patients with suspected invasive aspergillosis at a tertiary care hospital

**DOI:** 10.4102/ajlm.v15i1.3090

**Published:** 2026-10-14

**Authors:** Monika Devnani, Nazneen Pathan, Malvika Sharma, Aruna Vyas, Pooja Choudhary, Sidhya Choudhary

**Affiliations:** 1 Department of Microbiology, Sawai Man Singh Medical College, Jaipur, India

**Keywords:** invasive aspergillosis, galactomannan antigen, neutropenia, tuberculosis, HIV infection, fungal diagnostics, immunocompromised

## Abstract

**Background:**

Invasive aspergillosis (IA) is a major opportunistic fungal infection in immunocompromised patients. Serum *Aspergillus* galactomannan (GM) is an adjunctive marker in suspected IA.

**Objective:**

To determine the prevalence of serum GM positivity and associated risk factors among immunocompromised patients with clinical suspicion of IA.

**Methods:**

A cross-sectional observational study was conducted among 200 immunocompromised patients clinically suspected of IA at a tertiary hospital in Jaipur, India, from July 2023 to December 2023. Serum GM was tested using the Platelia™ *Aspergillus* GM enzyme immunoassay. Index values < 0.50 were considered negative, ≥ 0.50 positive, and 0.45–0.49 borderline. Demographic and clinical risk factors were recorded from clinical and laboratory records and analysed using chi-square test and multivariate regression.

**Results:**

Among 200 patients, 98 (49.0%) were GM positive, 92 (46.0%) negative, and 10 (5.0%) borderline. On multivariate analysis, neutropenia was the strongest independent predictor of GM positivity, followed by tuberculosis and lung disease. HIV infection was more frequent among GM positive patients but was not independently associated after adjustment.

**Conclusion:**

Serum GM positivity was high among immunocompromised patients with clinically suspected IA. Neutropenia was the strongest independent predictor of GM positivity, while tuberculosis and lung disease were also independently associated. Serum GM may aid evaluation when interpreted with clinical, radiological and microbiological findings. As confirmatory methods were not available for all patients, this study does not establish diagnostic accuracy of the assay.

**What this study adds:**

This study provides data on the prevalence of serum GM positivity among immunocompromised patients with suspected IA in Rajasthan, India. Neutropenia, tuberculosis and chronic lung disease were independently associated with GM positivity, supporting the use of serum GM assay in high-risk patients.

## Introduction

Invasive fungal infections are a growing concern in healthcare, particularly among immunocompromised individuals. Of these, invasive aspergillosis (IA) is one of the most severe, with a high risk of mortality if not diagnosed early.^1^ The causative organism, *Aspergillus* spp., particularly *A. fumigatus*, is ubiquitous and capable of causing deep-seated infections in vulnerable hosts including those undergoing chemotherapy, organ transplantation, prolonged corticosteroid use, or those with HIV infection, tuberculosis, and haematological malignancies.^2^ The burden of IA is particularly important in countries with a high prevalence of tuberculosis and HIV infection, as tuberculosis-associated lung damage and HIV-related immunosuppression are recognised risk factors for *Aspergillus* colonisation and invasive disease.^3^

Early diagnosis of IA remains challenging because clinical manifestations and radiological findings are often non-specific. Conventional methods such as fungal culture and histopathology are limited by invasiveness, low sensitivity, and delayed turnaround time.^1^ Consequently, non-culture-based biomarkers including serum and bronchoalveolar lavage galactomannan (GM) assays, beta-glucan detection, and *Aspergillus* polymerase chain reaction assays have gained importance in the evaluation of suspected IA.^4,5,6^

The *Aspergillus* GM assay detects polysaccharide antigens released during active fungal growth and has been incorporated into the European Organisation for Research and Treatment of Cancer/Mycoses Study Group Education and Research Consortium definitions as a mycological criterion for invasive fungal disease.^4^ However, interpretation of serum GM positivity may be influenced by host factors, antifungal exposure, concomitant infections, and false positive results.^4^

Despite increasing use of serum GM testing, limited data are available regarding the prevalence of serum GM positivity and associated clinical risk factors among immunocompromised patients with suspected IA in tertiary care settings in Rajasthan, India. The present study was therefore conducted to determine the prevalence of serum *Aspergillus* GM positivity and associated clinical risk factors in a tertiary care hospital setting.

## Methods

### Ethical considerations

The study was conducted following approval by the Institutional Ethics Committee of Sawai Man Singh Medical College and Hospital, Jaipur, Rajasthan, India, in April 2023 (Reference number 515/MC/EC/2023). Written informed consent was obtained from all participants or their legal guardians prior to sample collection. Patient confidentiality was maintained by anonymising all collected data and restricting access to study records to the investigators only.

### Study design and setting

This was a cross-sectional observational study conducted over a 6-month period (July 2023 – December 2023) in the Department of Microbiology at a tertiary care teaching hospital. The hospital serves as a referral centre, catering to a large number of immunocompromised individuals from both urban and rural settings.

### Study population and sampling

A total of 200 patients were recruited based on clinical suspicion of IA and the presence of one or more recognised host risk factors associated with IA.^4^ Clinical suspicion of IA was based on the presence of compatible clinical features such as persistent fever, respiratory symptoms, radiological suspicion of fungal infection, or underlying immunocompromised status prompting clinician-requested GM testing. The study employed purposive sampling of patients fulfilling the clinical and host risk factor criteria during the study period; participants were consecutively included until the desired sample size was achieved and to ensure inclusion of patients with varied immunosuppressive backgrounds, including neutropenia (< 1000 neutrophils/mm^3^ for > 10 days), haematologic malignancy, organ or stem cell transplant recipients, prolonged corticosteroid or immunosuppressant use, underlying lung disease (e.g. chronic obstructive pulmonary disease, asthma), HIV infection, or tuberculosis.^4^ Patients receiving antifungal therapy or recent beta-lactam antibiotics known to cause false-positive GM results were excluded.

### Specimen collection and laboratory testing

Peripheral blood samples were collected aseptically in a plain collection vial after obtaining informed consent and recording relevant clinical details according to the inclusion and exclusion criteria. Serum was separated by centrifugation and stored at 2 °C – 8 °C for a maximum of 7 days until testing. The Platelia™ *Aspergillus* GM enzyme immunoassay (Bio-Rad Laboratories, Gurugram, Haryana, India) was used, targeting *β*-1,5-galactofuranosyl epitopes of the GM antigen. The assay was performed according to the manufacturer’s instructions, and internal quality control procedures using kit-provided controls and calibrators were followed during assay performance.

### Test interpretation

An index value of < 0.50 was considered negative, while an index value of ≥ 0.50 was considered positive.^7^ Borderline results were defined as index values between 0.45 and 0.49 and were interpreted cautiously during analysis.

### Data collection and statistical analysis

Demographic and clinical data, including risk factors, were recorded in structured proformas. Data were analysed using SPSS version 25 (IBM Corp., Armonk, New York, United States). Associations between categorical variables and GM positivity were assessed using chi-square test. Multivariate logistic regression analysis was performed to identify independent predictors of GM positivity. Odds ratios (ORs) with 95% confidence intervals (CIs) were calculated. A *p*-value < 0.05 was considered statistically significant.

## Results

### Demographic profile of study participants

A total of 200 immunocompromised patients with clinical suspicion of IA were included in the study. Among them, 139 (69.5%) were male patients and 61 (30.5%) were female. The mean age of the participants was 45.4 ± 20.9 years, with an age range of 2 months to 89 years. The highest proportion of patients belonged to the 51–60-year age group.

### Galactomannan assay outcomes

Among the 200 patients, 98 (49.0%) were positive for serum GM antigen, 92 (46.0%) were negative, and 10 (5.0%) had borderline results (Table 1).

**TABLE 1:** Serum galactomannan assay results among study participants (*N* = 200) in Jaipur, India, between July 2023 and December 2023.

Result	*n*	%
Positive	98	49.0
Negative	92	46.0
Borderline	10	5.0

### Distribution of galactomannan positivity according to demographic characteristics

Among the 98 GM-positive patients, 66 (67.3%) were male and 32 (32.7%) were female. The highest GM positivity was observed in the 21–40-year age group, followed by the 41–60-year age group (Table 2).

**TABLE 2:** Distribution of galactomannan positivity according to demographic characteristics, in Jaipur, India, between July 2023 and December 2023.

Variable	GM positive		GM negative	
	*n*	%	*n*	%
**Gender**				
Male	66	67.3	69	75.0
Female	32	32.7	23	25.0
**Age group (years)**				
0–20	14	14.3	10	10.9
21–40	31	31.6	25	27.2
41–60	30	30.6	30	32.6
61–80	23	23.5	23	25.0
> 80	0	0.0	4	4.3

GM, galactomannan.

### Association of galactomannan positivity with clinical risk factors

Neutropenia was the most frequent host factor among the study population and was present in 97 of 98 GM-positive patients (99.0%) compared with 54 of 92 GM-negative patients (58.7%).

On univariate analysis, neutropenia showed the strongest association with GM positivity (OR: 68.26; 95% CI: 9.12–511.13; *p* < 0.001). Tuberculosis was also significantly associated with GM positivity (OR: 2.86; 95% CI: 1.20–6.84; *p* = 0.015). In addition, lung disease showed significant association with GM positivity (OR: 3.04; 95% CI: 1.28–7.23; *p* = 0.009) (Table 3).

**TABLE 3:** Univariate analysis of factors associated with galactomannan positivity, in Jaipur, India, between July 2023 and December 2023.

Variable	GM positive		GM negative		Odds ratio	95% CI	*p*-value
	*n*	%	*n*	%			
Male sex	66	67.3	69	75.0	0.69	0.36–1.30	0.245
Malignancy	13	13.3	4	4.3	3.36	1.06–10.73	0.031
HIV infection	15	15.3	8	8.7	1.90	0.76–4.71	0.163
Tuberculosis	21	21.4	8	8.7	2.86	1.20–6.84	0.015
Transplant history	2	2.0	6	6.5	0.30	0.06–1.52	0.124
Smoking	11	11.2	12	13.0	0.84	0.35–2.02	0.701
Lung disease	22	22.4	8	8.7	3.04	1.28–7.23	0.009
Neutropenia	97	99.0	54	58.7	68.26	9.12–511.13	< 0.001

GM, galactomannan; CI, confidence interval.

On multivariate logistic regression analysis, neutropenia remained the strongest independent predictor of GM positivity (adjusted OR: 94.69; 95% CI: 10.74–834.94; *p* < 0.001). Tuberculosis (adjusted OR: 5.98; 95% CI: 1.54–23.17; *p* = 0.010) and lung disease (adjusted OR: 4.27; 95% CI: 1.20–15.21; *p* = 0.025) also remained independently associated with GM positivity (Table 4).

**TABLE 4:** Multivariate logistic regression analysis of factors associated with galactomannan positivity, in Jaipur, India, between July 2023 and December 2023.

Variable	Adjusted odds ratio	95% CI	*p*-value
Male sex	0.91	0.42–1.97	0.806
Malignancy	4.17	0.98–17.69	0.053
HIV infection	1.73	0.61–4.91	0.299
Tuberculosis	5.98	1.54–23.17	0.010
Transplant history	1.40	0.14–14.35	0.775
Smoking	0.39	0.10–1.52	0.173
Lung disease	4.27	1.20–15.21	0.025
Neutropenia	94.69	10.74–834.94	< 0.001

CI, confidence interval.

HIV infection was more frequent among GM-positive than GM-negative patients, but was not independently associated with GM positivity after multivariate adjustment.

## Discussion

The present study demonstrated a high prevalence of serum GM positivity (49%) among immunocompromised patients with suspected IA in a tertiary care setting. Neutropenia emerged as the strongest independent predictor of GM positivity, while tuberculosis and chronic lung disease also remained independently associated after multivariate adjustment.

Among the clinical risk factors analysed, neutropenia showed the strongest association with GM positivity. This association is biologically plausible, as neutrophils play a central role in fungal clearance; their deficiency may result in increased fungal proliferation and antigenaemia. In this study, neutropenia was primarily secondary to malignancies, HIV infection and immunosuppressive therapy, all commonly encountered in this patient population. This may also reflect the referral pattern and high-risk composition of patients evaluated at this tertiary care centre. Neutropenia is a well-established risk factor, with several studies confirming that GM detection is particularly useful in neutropenic patients because of impaired clearance of fungal antigens.^4,7^

Tuberculosis and chronic lung disease also remained independently associated with GM positivity. The high regional burden of tuberculosis may have contributed to the observed association. Structural lung damage caused by tuberculosis and other chronic pulmonary diseases may predispose patients to *Aspergillus* colonisation and subsequent infection.^8^ These findings are consistent with previous studies reporting increased *Aspergillus* colonisation and GM positivity in patients with underlying pulmonary disease and prior tuberculosis.^9,10^

HIV infection was more frequent among GM-positive patients; however, it did not remain independently associated after multivariate adjustment. This suggests that coexisting pulmonary disease and other immunosuppressive conditions may have contributed to the observed association. Comparable observations have been reported in studies involving HIV-infected patients with advanced immunosuppression.^11,12^

Although other risk factors, such as solid organ transplantation and smoking, were recorded, they did not show statistically significant associations with GM positivity in this cohort.

The relatively high prevalence of serum GM positivity observed in the present study may reflect the highly selected immunocompromised population evaluated at a tertiary referral centre where patients often present with advanced disease and multiple underlying risk factors. In addition, the absence of standardised diagnostic classification and confirmatory microbiological or radiological evaluation in all patients may have contributed to inclusion of patients with *Aspergillus* colonisation rather than proven invasive disease. Therefore, serum GM positivity should be interpreted cautiously, as false-positive results may occur because of fungal colonisation, cross-reactivity, certain antimicrobial agents, and dietary or environmental factors.^1,4^

### Limitations

This study had several limitations. It was conducted at a single tertiary care centre with a relatively limited sample size, which may affect the generalisability of the findings. In addition, the study population was largely composed of high-risk immunocompromised patients referred to a tertiary care setting, which may have contributed to selection bias and the relatively high prevalence of serum GM positivity observed in this study. The cross-sectional study design did not permit assessment of longitudinal clinical outcomes, treatment response, or mortality.

The diagnosis of IA was not established using standardised European Organisation for Research and Treatment of Cancer/Mycoses Study Group Education and Research Consortium criteria, and confirmatory diagnostic methods such as fungal culture, histopathology, bronchoalveolar lavage GM testing, or *Aspergillus* polymerase chain reaction were not available for all patients. Radiological correlation was also not systematically performed in all cases. Therefore, the study evaluated the prevalence of serum GM positivity among clinically suspected cases rather than confirmed IA, and the possibility of *Aspergillus* colonisation or false-positive GM results could not be completely excluded.

Detailed immunological parameters such as CD4 counts in HIV-infected patients were not uniformly available for analysis, and tuberculosis was not subclassified into active or previously treated disease. Borderline GM results were not analysed as a separate diagnostic category, and repeat sampling or longitudinal follow-up of patients with borderline results was not feasible in this high-volume tertiary care setting, limiting assessment of the clinical significance of these findings.

Because confirmatory diagnostic classification was unavailable, diagnostic accuracy parameters such as sensitivity, specificity, positive predictive value, and negative predictive value were not calculated. Further prospective multicentric studies incorporating standardised diagnostic criteria and comprehensive microbiological, molecular, and radiological evaluation are required to better define the clinical utility of serum GM testing in immunocompromised patients.

### Conclusion

A high prevalence of serum GM positivity was observed among immunocompromised patients with suspected IA in this study. Neutropenia emerged as the strongest independent factor associated with GM positivity, while tuberculosis and chronic lung disease also showed significant independent association.

These findings highlight the potential role of serum GM testing as part of the diagnostic evaluation in high-risk immunocompromised patients. However, GM positivity should be interpreted cautiously and in conjunction with clinical, radiological, and other microbiological findings because false-positive results may occur.

Further prospective multicentric studies incorporating standardised diagnostic criteria and confirmatory methods are required to better establish the clinical utility of serum GM testing in IA.
